# Pyrethroid and Chlorpyrifos Pesticide Exposure, General Intellectual Abilities, and Executive Functions of School Children from Montevideo, Uruguay

**DOI:** 10.3390/ijerph20075288

**Published:** 2023-03-28

**Authors:** Danelly Rodríguez, Gabriel Barg, Elena I. Queirolo, James R. Olson, Nelly Mañay, Katarzyna Kordas

**Affiliations:** 1 Department of Epidemiology and Environmental Health, University at Buffalo, Buffalo, NY 14214, USA; danellyr@buffalo.edu (D.R.);; 2 Department of Neuroscience and Learning, Catholic University of Uruguay, Montevideo 11600, Uruguay; 3 Department of Pharmacology and Toxicology, University at Buffalo, Buffalo, NY 14214, USA; 4 Faculty of Chemistry, University of the Republic of Uruguay (UDELAR), Montevideo 11200, Uruguay

**Keywords:** pyrethroids, chlorpyrifos, pesticide exposure, cognition, child health, executive function

## Abstract

Children’s developing brains are susceptible to pesticides. Less is known about the effect of exposure to chlorpyrifos and pyrethroids on executive functions (EF). We measured urinary 3,5,6-trichloro-2-pyridinol (TCPy), a metabolite of chlorpyrifos, and urinary 3-phenoxybenzoic acid (3-PBA), a general, nonspecific metabolite of pyrethroids in first-grade children from Montevideo, Uruguay (*n* = 241, age 80.6 ± 6.4 months, 58.1% boys). EFs were assessed with the Intra-dimensional/Extra-dimensional shift (IED), Spatial Span (SSP), and Stockings of Cambridge (SOC) tests from the Cambridge Neuropsychological Test Automated (CANTAB) Battery. General intellectual ability (GIA) was assessed using the Woodcock–Muñoz Cognitive battery. Median (range) urinary TCPy and 3-PBA levels were 16.7 (1.9, 356.9) ng/mg of creatinine and 3.3 (0.3, 110.6) ng/mg of creatinine, respectively. In multivariable generalized linear models, urinary TCPy was inversely associated with postdimensional errors on the IED task β [95% CI]: −0.11 [−0.17, −0.06]. Urinary 3-PBA was inversely associated with the total number of trials −0.07 [−0.10, −0.04], and the total number of errors −0.12 [−0.18, −0.07] on the IED task. When TCPy and 3-PBA were modeled together, the associations did not differ from single-metabolite models. We found no evidence of effect modification by blood lead level (BLL). Pesticide exposure may affect EF performance in urban children.

## 1. Introduction

Exposure to pesticides is a health concern globally, especially in countries in Latin America and the Caribbean [1]. For more than a decade, Uruguay has had one of the highest pesticide uses per hectare in Latin America, with peaks in 2010–2014 [2]. Children are especially sensitive to the neurotoxic consequences of environmental contaminants, including pesticides [3]. Numerous cohort studies in the rural and urban regions of the United States (U.S.) have described associations of prenatal exposures to pyrethroids and organophosphates (OPs), including chlorpyrifos, with poor IQ in 3–11-year-old children [4,5,6,7,8,9,10,11,12]. Similarly, studies in rural regions of Latin America have reported associations of prenatal exposures to organochlorine pesticides with developmental motor delays in Mexican and Ecuadorian 2-year-old children [13,14,15,16]. A followup study suggested that exposures to organochlorines in utero were associated with deficits in IQ, spatial orientation, and quantitative and verbal skills [17,18,19].

Although extensive evidence suggests that prenatal exposure to pesticides impacts IQ across childhood, there is an evidence gap in understanding the links between childhood pesticide exposure and executive functions (EF). Cross-sectional studies conducted in Latin America found relationships between exposure to OPs and EFs such as working memory, visual perception, selective attention, and language in school-age children [20,21,22,23]. However, these studies were based on measures of household proximity to agricultural crops, rather than biomarkers (i.e., metabolites) of exposure.

Studies that examined urinary biomarkers of childhood OP exposure, found inverse associations between urinary dialkyl phosphates (DAP) and EF domains, including working memory [24] and cognitive flexibility [25] in 6–7-year-old children. However, few studies have investigated exposure to chlorpyrifos and pyrethroids, which continue to be widely used, and aspects of children’s cognition. Even fewer of these studies measured specific metabolites of chlorpyrifos or pyrethroid exposures in children or assessed the effects of multiple pesticides together on cognition. Because of its cost-effectiveness, chlorpyrifos use is ubiquitous. The prevalence of chlorpyrifos use on crops worldwide is in the 5–39% range [26]. During the last decade, pyrethroids use has also increased to replace other more toxic pesticides and represents 38% of the world insecticide market [27]. In Uruguay, 38,000 tons of pesticides (pyrethroids and chlorpyrifos among others) were produced in 2012 [2]; many of these present high levels of toxicity, and more than 85% of the active ingredients used are imported [2].

One study in Costa Rica discovered that urinary 3,5,6-trichloro-2-pyridinol (TCPy), a specific metabolite of chlorpyrifos, and 3-phenoxybenzoic acid (3-PBA), a general biomarker of exposure to pyrethroids, was associated with deficits in processing speed and memory, as measured by the Wechsler Intelligence Scale (WISC-IV), in 6–9-year-old children [28].

In addition to gaps in exposure assessment, studies have paid less attention to specific neurodevelopmental domains such as EFs, and very few studies thus far have included children from urban areas. To address these gaps, we examined the cross-sectional association of urinary TCPy and urinary 3-PBA with general intellectual ability (GIA) and EFs of urban school children from Montevideo, Uruguay. We hypothesized inverse associations between urinary TCPy and 3-PBA and the cognitive performance measures.

Additionally, previous studies have indicated that lead exposure, even at lower levels, is linked to EF deficits in children [29,30]. Because lead, like pesticides, is a ubiquitous neurotoxicant and remains a public health problem in Uruguay [23,24,25], we investigated the extent to which children’s blood lead levels (BLLs) modify the association between pesticide exposure and tests of cognitive function. We hypothesized that the effect of pesticide exposure on cognitive performance will be higher in children with BLL ≥ 3.5 μg/dL compared to those with levels below 3.5 μg/dL.

## 2. Materials and Methods

### 2.1. Setting and Recruitment

The present study is a post hoc analysis of data from the Salud Ambiental Montevideo (SAM) study, originally designed to examine metal exposure, including arsenic, cadmium, and manganese among school-age children in Montevideo, Uruguay [31,32,33,34,35,36]. A total of 357 first-grade children out of 673 eligible children were recruited from elementary schools in locations with suspected or confirmed metal contamination, as previously described [36,37]. Children and parents completed questionnaires, neuropsychological tests, and biological sampling of urine and blood. Research ethics boards approved the study protocols at the Pennsylvania State University, the Catholic University of Uruguay, the Faculty of Chemistry at the University of the Republic of Uruguay, and the University at Buffalo. Of the enrolled 357 children, 116 did not have data on urinary biomarkers or full cognitive evaluation, which resulted in a study sample of 241 children.

### 2.2. Sociodemographic Measures

The caregivers, usually mothers, reported demographic information about themselves, their children, and their household environment. Characteristics for children included sex, age, and medical history. A social worker assessed the developmental environment of the household during a home visit using a Spanish version of the Home Observation for Measurement of the Environment (HOME) Inventory [38]. The HOME inventory score sums across 59 items related to parental accountability, motivation, emotional environment, learning materials and opportunities, active stimulation, household participation, parental involvement, and physical surroundings.

### 2.3. Analysis of Pesticide Metabolites in Urine

First void urine samples were collected by children with the assistance of caregivers and brought to school in collection cups provided for this purpose. The samples were kept at −20 °C until shipment to the U.S. Pyrethroid and chlorpyrifos metabolite analysis was completed at the Analytical Toxicology Laboratory, University at Buffalo. One metabolite of chlorpyrifos and 4 metabolites of pyrethroid pesticides were analyzed. The chlorpyrifos-specific metabolite, TCPy, was analyzed by gas chromatography–mass spectrometry (GC/MS) using chemical ionization in negative ion mode, with 13C-15N-3,5,6-TCPy as an internal standard, as described previously [39]. The general pyrethroid metabolite, 3-PBA, along with the alpha-cypermethrin (αCM)-specific metabolite, cis-3-(2,2-dichlorovinyl)-2,2-dimethyl-(1-cyclopropane) carboxylic acid (cis-DCCA), and the lambda-cyhalothrin (λCH)-specific metabolite, lambda cyhalothric acid, (λ-CA), were quantified in urine by negative ion chemical ionization by GC/MS, as described previously. In addition to cis-DCCA, trans-DCCA (trans 3-(2,2-dichlorovinyl)-2,2-dimethylcyclopropane carboxylic acid) was also measured since both are metabolites of cypermethrin [40]. Limits of detection (LOD) were as follows: TCPy (0.22 ng/mL), 3-PBA (0.45 ng/mL), cis-DCCA (0.09 ng/mL), trans-DCCA (0.18 ng/mL), and λCA (0.11 ng/mL).

Creatinine concentration in urine was measured at the Analytical Toxicology Lab, University at Buffalo, using the Jaffe reaction [41]. The urinary concentrations of pesticide metabolites were expressed as ng/mL urine (wet weight) and ng/mg creatinine (creatinine adjusted), according to the following formula:

Creatinine adjusted pesticide level (ng/mg creatinine) = [pesticide metabolite (ng/mL urine)] [creatinine (mg creatinine/mL urine)]

### 2.4. Lead and Hemoglobin in Blood

A 25-gauge safety butterfly blood collection set (Vacutainer, Becton Dickinson, Franklin Lakes, NJ, USA) was utilized to collect blood samples. Fasting venous blood was collected in a serum tube with a clot activator and separator gel (Becton Dickinson, Franklin Lakes, NJ, USA). A drop of venous blood was extracted from the serum tube instantly and used to measure hemoglobin with a portable hemoglobinometer (HemoCue Inc., Lake Forest, CA, USA). Quality control was conducted daily with standard controls (low, medium, high). A second blood sample was collected into a heparin-coated tube (Vacutainer, Becton Dickinson, Franklin Lakes, NJ, USA). Blood samples were then stored on ice and transported to the Toxicology Laboratory “CEQUIMTOX” (Specialized Center for Chemical Toxicology) of the Faculty of Chemistry, University of the Republic of Uruguay. CEQUIMTOX participates in the Center for Disease Control’s (CDC) Lead and Multi-Element Proficiency Program (LAMP) and the Interlaboratory Program of Quality Control for Lead in Blood, in Spain (PICC Pb-S).Whole blood samples were analyzed by Atomic Absorption Spectrometry (AAS, VARIAN SpectrAA-55B) using flame (FAAS) or graphite furnace (GFAAS) ionization methods, depending on the volume of whole blood available. The graphite furnace was used for blood samples with volumes below 2 mL. In 2009–2010, the LOD was 2.5 µg/dL for FAAS and 2.0 µg/dL, then 0.8 µg/dL for GFAAS. Subsequently, in 2011–2013, the FAAS LOD was 1.8 µg/dL, and GFAAS was 0.8–1.0 µg/dL.

### 2.5. Woodcock–Muñoz Tests of General Cognitive Abilities

The Woodcock–Muñoz test battery is a Spanish version of the Woodcock–Johnson battery [42,43] that is typically utilized to assess cognitive impairment in a clinical environment [44,45,46]. Seven tests in the cognitive battery were conducted: verbal comprehension, visual–auditory learning, spatial relations, sound blending, concept formation, visual matching, and reversed numbers. Each subtest yields a sex- and age-standardized score, called the W score, as described previously [34]. General intellectual ability (GIA) was measured as a weighted composite of the 7 test scores. The median internal consistency reliability for all ages is 0.87 [47].

### 2.6. Cambridge Neuropsychological Test Automated Battery (CANTAB)

The CANTAB test is a battery of cognitive assessments given on a touchscreen device that has utility in neurotoxicology research [48,49,50]. CANTAB instructions were given to the children in Spanish by the study researcher. Because the CANTAB scales administered in this study use nonverbal, visual, and auditory stimuli, no language translation was required. Three CANTAB tests were used to assess executive functions: Stockings of Cambridge (SOC), the Intra-dimensional/Extra-dimensional shift task (IED), and Spatial Span (SSP). Among adult populations, reliability estimates are appropriate for the IED (ICC = 0.79) and moderate for the SOC and SSP tasks (ICC = 0.60 and 0.68, respectively) [51]. However, test–retest reliability assessments among children have not been conducted. In one study, intraclass correlations (ICC) for the IED were 0.78–1.0 in children and adolescents ages 11–17 with ADHD [52].

The SOC measures spatial planning capacity. In this task, children viewed 2 colored balls within 3 “stockings.” One stocking display was in the middle, and the second one was at the top of the screen. Children were asked to recreate a third stocking display at the top of the screen by putting balls in their chosen place, 1 at a time. Movement of the balls at the bottom of the stocking was not permitted before the ball on the top was moved. Four problems were displayed, and the number of moves and complexity increased progressively with trials. Spatial planning was measured as the number of problems that were correct with the minimum number of moves.

The IED task is an assessment of cognitive flexibility (rule acquisition and reversal). 2 dimensions were displayed: pink shapes and white lines. Children were instructed to click on the dimension they thought was correct. Implicit rules were applied, and the children learned these as responding proceeds. Sound and color feedback from the computer alerted the children as to whether their choice was correct. A total of 9 stages were administered. In the IED, stages 1 and 2 consisted of shapes. After a couple of trials, the rule regarding which pink shape was correct changed. Once children learned the new rule, the responding continued with more trials. In stages 3, 4, and 5, white lines were added on the shapes. Stages 6 and 7 introduced new shapes and new white line patterns, considered the intra-dimensional shift. The white lines became the important stimuli at stages 8 and 9, constituting the extra-dimensional shift. The IED outcomes of interest were estimated as the number of stages finished, the number of trials completed, the number of errors, and the pre-and post-extra-dimensional shift errors. In scoring children’s performance, an adjustment approach from the CANTAB Administration Guide was used to adjust the total number of errors and trials. Specifically, as in previous research [52,53], 25 errors were counted per failed stage because children that advanced to more stages could make more errors. With this scoring strategy, 5 children failed the IED test prior to stage 4. Two children were assigned zero errors after they failed to complete stage 9. The values for these 7 children were replaced with errors missing on the pre-and post-extra-dimensional shift. The SSP is an assessment of short-term visual memory, where 10 white squares are randomly displayed on a computer screen. The 10 squares change color 1 at a time. Children were asked to touch the boxes, remembering the sequence of color changes. As the test progressed, the number of boxes increased from 2 to 9. Thus, working memory was measured as span length (2–9 boxes). Other outcomes included errors, the number of attempts, and latency to response. In our study, 27 children (8%) failed to replicate the pattern during the 2-square trial after 3 tries and were assigned a maximum span length of 1.

### 2.7. Statistical Methods

Statistical analyses were conducted with SAS version 9.4 (SAS Institute Inc., Cary, NC, USA). Summary statistics for sociodemographic, biological, and cognitive measures included means (standard deviations) or medians (ranges), depending on the distribution. Biomonitoring data from the National Health and Nutrition Examination Survey (NHANES) 2007–2010 were utilized to compare exposure distributions between the study children and similarly aged children in the U.S.

Urinary pesticide biomarker values under the detection limit were assigned LOD/$\sqrt{2}$. Then, values were normalized to creatinine excretion. Finally, due to non-normal distribution, these variables were natural log (ln)-transformed for further analysis. The 4 urinary pyrethroid metabolites were highly correlated (Supplemental Table S1). Therefore, we selected 3-PBA, the general biomarker of pyrethroids, for further statistical analysis. To conduct stratified models by BLL, described below, the children were split into 2 groups based on whether their BLLs fell <or were ≥3.5 µg/dL.

We compared participant characteristics between the analytical sample (those with complete cognitive performance and urinary biomarker data) and the children excluded from the study (Table 1). Multiple imputation (MI) of demographic characteristics was conducted to employ the sample of 241 that had complete data on exposure and outcome measures. Imputation was implemented in SAS across 50 cycles, and commands were selected for each variable to match its distribution (i.e., normal or Poisson). Using imputed data, separate and combined (TCPy and 3-PBA in the same model) generalized linear models were fitted to test the associations between each ln-transformed creatinine-adjusted pesticide metabolite and each cognitive endpoint. An identity log or link function was added using a generalized estimation equation to address school clusters. The reported beta coefficients in our models represent a change in a cognitive endpoint for every 1-unit change in ln-transformed pesticide urinary concentration.

Intraclass correlations for the cognitive outcomes within schools ranged 0.06–0.16, and clustering by school was considered in the models. Multivariable models included age, sex, the HOME score, season of the year in which the assessment was conducted, the BLL category, and blood hemoglobin as covariates. These variables were selected based on prior literature [32,33,34], followed by covariate retention based on a > 10% difference between the crude and adjusted associations. In addition, we conducted stratified analyses to assess effect modification by BLL category (< or ≥3.5 µg/dL). We applied the Holm–Bonferroni sequential procedure [54] to all analyses to adjust for familywise error rate inflation.

## 3. Results

### 3.1. Sample Characteristics

Table 1 shows sample characteristics, including GIA and EF scores, for children with complete urine and cognitive data compared to children excluded from the analysis. The complete sample had more males (58.1% vs. 41.9%), was about 2 months younger, and completed more trials on the IED task. No other differences were noted.

### 3.2. Comparisons of Urinary Pesticide Metabolite Concentrations among Uruguayan and U.S. Children

Table 2 presents the distributions of all pesticides in Uruguayan children with complete urine and cognitive data (*n* = 241) compared to similarly aged children in the U.S. around the same period. The geometric mean urinary levels of TCPy and 3-PBA were roughly an order of magnitude higher in Uruguayan children compared to 6–11-year-old children in the U.S. At the 95th percentile, urinary concentrations of trans-DCCA were about 3-fold higher in Uruguayan children compared to U.S. children during 2007–2009 and about 1.4-fold higher in Uruguayan children compared to U.S. children during 2009–2010.

### 3.3. Association between Urinary TCPy, 3-PBA, and Cognitive Function Tests

Table 3 presents the associations between ln-transformed TCPy and 3-PBA and cognitive endpoints following multiple imputation of missing data, and with the Holm–Bonferroni correction. There were no statistically significant associations between GIA scores and pesticide metabolite concentrations. Regarding IED endpoints, there were statistically significant inverse associations between 3-PBA with the total number of trials completed in the IED task, 3-PBA with the total number of errors, and TCPy with the total number of extra-dimensional stage errors. When the 2 urinary biomarkers were modeled together, 3-PBA continued to be associated with total trials and total errors on the IED task. On the other hand, TCPy was no longer associated with postdimensional errors on the IED task. Although not reported, crude models were consistent with adjusted models.

### 3.4. BLL as an Effect Modifier

Table 4 shows the results of analyses stratified by BLL< or ≥3.5 μg/dL. The association between urinary TCPy and 3-PBA and cognitive performance did not differ among children with BLLs of <3.5 μg/dL and those with ≥3.5 μg/dL as evidenced by overlapping confidence intervals.

### 3.5. Sensitivity Analyses

We conducted a sensitivity analysis among the nonimputed sample (*n* = 222). We repeated our main analysis and, for the most part, results remained unchanged (Supplemental Table S2). Upon the Holm–Bonferroni correction, statistical significance was reached for inverse associations between TCPy, the total number of postdimensional errors 3-PBA, the total number of trials completed, and errors on the IED task. As in models with imputed data, results remained unchanged when TCPy and 3-PBA were modeled together (Supplemental Table S2).

## 4. Discussion

In contrast to the strong evidence that prenatal pesticide exposure affects children’s cognitive development, questions remain on how childhood exposure to commonly used pesticides like chlorpyrifos and pyrethroids affect cognitive performance, including EFs. In this cross-sectional analysis, we investigated the association between 2 urinary metabolites indicative of pesticide exposure with general cognitive abilities and EFs among ~7-year-old children from Montevideo, Uruguay. Urban children exposed to pyrethroids, individually and in combination with chlorpyrifos, completed fewer trials on rule acquisition and reversal tests on the IED (cognitive flexibility) task. However, it is important to point out that the effect sizes for these associations were small. Lead continues to be a toxicant of concern globally and has similar clinical manifestations to pesticides, including neurodevelopmental deficits [55,56]. In addition to accounting for lead exposure in our main analysis, we explored effect modification of BLL (≥3.5 vs. <3.5 μg/dL) on the associations between urinary pesticide concentrations and each cognitive endpoint. We found no evidence of effect modification by BLL. Although not statistically significant, our findings of negative association between both pesticide biomarkers and general cognitive abilities are similar to previous cohort studies on prenatal exposures to OP pesticides (measured as urinary biomarkers DAP and TCPy), or pyrethroids (3-PBA) and global measures of IQ in school-age children [4,5,6,7,8,9,10,11,12].

Pesticide use in Latin America and the Caribbean accounts for 20% of global consumption [2], but these regions lack regulations or rigorous implementation compared to the U.S. This may be a reason for higher exposure in this region [57] and the differences in biomarker concentrations between U.S. and Uruguayan children of similar ages. For example, pesticide metabolites were up to 10-fold higher in Uruguayan children than in the general U.S. population of 6–11-year-olds. During the last 20 years, the growing use of pesticides in Uruguay has been linked to technological innovations and the expansion of agriculture, especially that associated with soybean cultivation [58]. In 2012, pesticide use peaked in Uruguay. Specifically, 38,000 tons of pesticides were produced [2], and more than 85% of the active ingredients were imported [2].

Several cross-sectional studies in Latin America found associations between residential distance to crops and EFs [20,21,22,23], indicating that pesticide exposure from agricultural operations is harmful to children’s neurodevelopment. However, although we did not have information regarding sources of exposure, the children in our study live in a large metropolitan area and it is likely that exposures are at least in part linked to pesticide residues on food [59,60,61]. We are also aware that residential pesticide use indoors is common in urban populations [62], and it is a possible source of exposure, although we have not quantified such pesticide use among our study participants. Although sources of exposure in our study population compared to other regions of Latin America may differ, pesticide metabolite levels measured in our study are comparable to levels reported in studies that were conducted in 2007 for children living in agricultural regions, where children experience long-term exposure. For example, median urine samples of children living in rural agricultural regions of Nicaragua had lower median levels of TCPy (3.7 vs. 16.76 ng/mg) and 3-PBA (2.8 vs. 3.30 ng/mg) metabolites than those found in our study [63]. Similarly, children from rural Costa Rica had lower median levels of TCPy (1.4 vs. 16.76 ng/mg) and 3-PBA (0.8 vs. 3.30 ng/mg) [28]. Our study was conducted in 2009–2013, when pesticide use in Uruguay had increased [2]. Thus, the comparative exposure patterns and levels in Uruguay may be due to changes in pesticide use over time and indicate that chronic pesticide exposure is a significant public health concern in cities. Furthermore, emerging research indicates that urban populations may be more likely exposed to pesticides than rural populations. Specifically, in some studies, Hispanic/Latino children living in cities had a higher prevalence of pyrethroid pesticides than those from rural towns (75.0% vs. 57.5%) [64,65], highlighting the need for research on urban populations.

Regarding the link between biomarkers of pesticide exposure and cognitive performance in children, our results are consistent with a cross-sectional study in 7-year-old children that found an inverse association between DAPs, a urinary biomarker of OP exposure, and the Wisconsin Card Sorting Test (WCST), a measure of cognitive flexibility [25]. Although we did not observe associations with other domains of EFs, other cross-sectional studies found associations between urinary biomarkers, including DAPs, TCPy, and 3-PBA and working memory in 5–6-year-old children [10,24,28]. Different domains of EFs have distinct developmental trajectories. Specifically, cognitive flexibility has a crucial period of development that ranges from 7 to 9 years of age [66]. On the other hand, attentional control emerges during infancy and develops rapidly in early childhood [66]. In the context of the timing of neurodevelopment, it is possible we saw an association specifically with cognitive flexibility because the 7–8-year-old children in our sample were experiencing growth in this domain. Because EFs continually mature over time [66], it is important to study EF domains at distinct ages.

The brain’s prefrontal cortex plays a vital role in the neural mechanisms underlying EFs [67]. The mechanistic paths by which chlorpyrifos and pyrethroids can harm children’s neurocognitive development are not understood. However, the associations described in epidemiologic studies for both pesticide metabolites may be explained by animal studies. Chlorpyrifos degrades into oxon metabolites that block acetylcholinesterase enzymes responsible for breaking down acetylcholine (AChe) [68]. AChe is a chief neurotransmitter that is essential in modulating cognitive function. Neurodevelopmental impacts of nonorganophosphate pesticides, such as pyrethroids, have been studied less in humans. Animal studies suggest that pyrethroids affect the nervous system by interrupting sodium channels in axonal membranes of neurons and inducing neuronal apoptosis [69]. Furthermore, animal studies have revealed that early life exposure to pyrethroids diminishes muscarinic cholinergic receptors, thereby hindering the dopaminergic system [70]. Another possible explanation for our findings is that among humans, hindered dopaminergic activity in the prefrontal cortex is associated with EF impairment, specifically in the domains of attention and cognitive flexibility [70]. Oxidative stress is another hypothesized mechanism for the neurotoxicity of chlorpyrifos and pyrethroids. Neurons are susceptible to oxidative stress [71] due to their higher-fat content, low antioxidative capabilities, and higher oxygen demands [72]. These critical neuronal processes underlying synaptic proliferation underpin the maturation of the prefrontal cortex [73], the regions that support the development of EFs [74,75].

We found an inverse association between TCPy and postdimensional errors, which was not in the direction that we hypothesized. The IED is a complex task that measures cognitive flexibility and entails learning when the implied rule of the test shifts (i.e., when the descriptors/characteristics of the accurate response change). Following this shift, the task is relatively more complex than before. The criterion for understanding every rule is built on successive responses, and if a child fails to learn after 50 trials, the test terminates. IED responses may depend on the development of the prefrontal cortex and EFs [76]. For example, IED performance in 7-year-old children is poorer compared to older children [76]. Therefore, optimal performance is likely to improve first in tasks assessing pre-executive shifts. We used pre-executive shift errors as a confounder in regression models to further explore the observed associations. The results remained unchanged (results not shown), suggesting that errors made prior to the extra-dimensional shift did not influence the total trials or total errors on the IED task. It is likely that due to the age of the children in this study, the task could have been challenging to most children post-shift, regardless of pesticide exposure.

The present study offered several strengths. First, pesticide exposures were estimated using biomarkers in urine. Unlike urinary biomarkers of exposure, residential distance used in previous studies [20,21,22,23] does not reflect all routes of exposure or the specific pesticides absorbed by the body. Compared to external/environmental measures of contaminants, biomarkers are believed to be more directly related to adverse health effects [59]. Biomonitoring is the gold standard for exposure assessment because it reflects all sources and routes of exposure, uptake, absorption, and metabolism [77]. Second, although there are no previous investigations to which we could compare our findings, our study sought to build a foundation for understanding if coexposure to lead may result in poorer performance than pesticide exposure alone. Associations between lead and EFs have been well established [78] and it is important to consider effect modification by lead in future studies. Third, to our knowledge, we are the first study to assess the relationship between pesticides and EFs using the CANTAB. This test battery has some advantages over standardized clinical tests, including measuring different domains of EFs and robust data relating specific performance deficits to focal brain aberrations [49]. Many tests in the CANTAB use nonverbal cues, meaning they can be given in any geographical area regardless of the language. Because of its computerized nature, the CANTAB scores are slightly affected by variations among testers, and the administration is less cumbersome than previously used measures like the Weschler Intelligence Scale (WISC-IV) and Wisconsin Sorting Tasks. Fourth, we utilized a comprehensive set of covariates that were chosen a priori [32,33,34,35] and further selected based on a >10% difference between the crude and adjusted associations. In contrast to most studies on childhood exposures to pesticides [10,22,23,25,28], we adjusted our models for iron status (blood hemoglobin) and the HOME Inventory. Iron deficiency [79,80] and the HOME Inventory [34] are salient predictors of cognitive performance in children. Fifth, we accounted for the effect of hydration status on urinary metabolite excretion by adjusting for urinary creatinine [81]. Sixth, to compensate for missing covariate data, we conducted our analysis in a sample attained using multiple imputation [82,83]. Furthermore, we conducted sensitivity analyses in a complete case sample. Sensitivity results remained the same as the main results, thereby increasing confidence in the observed inferences.

This study also had limitations. First, we had a modest sample size which limited our statistical power and resulted in wide confidence intervals. This limited our ability to make definitive conclusions on the associations between pesticide exposure and EFs in children. Second, we measured pesticide metabolites in single spot urine samples, without repeated measurements over time. Pesticide metabolites have relatively short biological half-lives, ranging 6.4–27 h [84]. While our measures indicate that the study children experienced recent exposure to both pesticides, without repeat sampling, we are unable to determine whether urinary metabolite concentrations in this study represent longer-term exposures. We assume, but cannot corroborate, that children were chronically exposed to pesticides due to the relatively high urinary concentrations as well as the extensive use of pesticides during the study years 2010–2014 [2]. In addition, prenatal exposure patterns or levels are unknown for our study sample. Another limitation is that measures of performance on neuropsychological tests alone may not detect subtle exposure-induced impacts on neural function. For example, a study did not find significant differences in cognitive flexibility test performance in adolescents with prenatal pesticides exposure but observed a concurrent decrease in prefrontal cortex activation (measured with near-infrared spectroscopy) [85]. Combining neuroimaging studies with neuropsychological assessment could be a direction for future research. Finally, this was a cross-sectional study, which limited our ability to infer causal relationships and estimate risk of poor cognitive outcomes due to pesticide exposure.

This study and its findings suggest several directions for future research, including a focus on longitudinal assessment of childhood pesticide exposures and their impact on EFs into adolescence. Future studies should identify sources of pesticide exposure in urban populations as well as assess genetic or other factors related to pesticide metabolism to further account for differences in metabolite concentrations in urine and their relationship with neurodevelopmental outcomes. It has been well established that different paraoxonase-1 (PON1) polymorphisms affect neurodevelopment in children exposed to OP pesticides [86,87]. Some studies have linked pesticide exposures with lower AChe activity [88,89] in school children from Ecuador. Thus, AChe could serve as a biomarker of neurological effects of pesticide exposure in children.

## 5. Conclusions

We found evidence that childhood exposure to chlorpyrifos or pyrethroids are associated with specific tasks related to executive function but not general cognitive abilities in urban children aged ~7 years. Lead did not modify these relationships. Longitudinal studies with repeated measures are necessary to understand the potential cognitive effects associated with pesticide exposure, which may motivate the phasing out of specific pesticides that are hazardous to human health and assist in the creation of new guidelines for the use of pesticides in urban areas.

## Figures and Tables

**Table 1:** A comparison of demographic characteristics and cognitive performance between Montevideo children aged ~7 years with urine and cognitive data (included in the study) and those without urine and cognitive data (excluded) (*n* = 241).

Variables	N	Included	N	Excluded
Female	101	41.9%	57	50.9%
Male	140	58.1%	55	49.1%
Age, months	241	80.6 ± 6.4	110	82.7 ± 6.2
HOME Inventory score	235	44.6 ± 8.4	97	44.6 ± 7.2
Blood lead levels, µg/dL	231	4.2 ± 2.1	84	4.0 ± 2.0
Hemoglobin, g/dL	236	13.2 ± 1.1	86	13.2 ± 1.1
General intellectual ability	241	90.2 ± 16.4	98	87.2 ± 17.6
SSP, span length	241	3.6 ± 1.0	97	3.5 ± 1.2
SSP, total errors	241	10.4 ± 4.1	97	10.7 ± 4.6
SOC, problems solved	241	5.0 ± 1.8	98	5.0 ± 1.8
IED, total trials	241	146 (64–386)	101	141 (68–425)
IED, stages completed	241	8 (2–9)	101	7 (1–9)
IED, total errors	241	54 (8–181)	101	56 (9–222)
IED, predimensional errors	241	7 (3–40)	101	7 (3–47)
IED, postdimensional errors	241	21 (0–36)	101	23 (0–35)

Categorical variables are presented as N and %, continuous variables presented as Mean ± SD, continuous variables, continuous variables that were not normally distributed presented as Median (range); Abbreviations: SSP: Spatial Span, SOC: Stockings of Cambridge, IED: Intra-Extra Dimensional Set Shift.

**Table 2:** A comparison of urinary pesticide metabolite concentrations (ng/mg creatinine) among children with complete data (*n* = 241) aged ~7 years from Montevideo with children aged 6–11 years from the U.S. population.

Pesticide Metabolite	N	Geometric Mean	P50	P75	P95	LOD	#<LOD ^b^
SAM children							
TCPy	241	17.84	16.76	27.09	60.85	0.22	0
3-PBA		3.44	3.30	5.53	11.60	0.45	1
λCA		0.13	0.08	0.18	0.72	0.11	139
cis-DCCA		0.82	0.81	1.51	3.44	0.09	2
trans-DCCA		2.91	2.90	4.97	12	0.18	1
NHANES Child Biomonitoring Data (6–11 years old)							
TCPy (2007–2008)	385	1.72	1.85	3.18	6.04	0.10	-
TCPy (2009–2010)	386	1.12	1.46	2.38	5.81		-
3-PBA (2007–2008)	371	0.39	0.36	1.13	9.88	0.10	-
3-PBA (2009–2010)	383	0.55	0.48	1.43	8.51		-
Trans-DCCA (2007–2009) ^a^	384	-	-	-	4.01	0.60	-
Trans-DCCA (2009–2010) ^a^	386	-	-	-	8.64		-
Cis-DCCA (2007–2008) ^a^	384	-	-	-	-	0.50	-
Cis-DCCA (2009–2010) ^a^	386	-	-	-	-		-

^a^ Geometric mean and median not calculated, the proportion of results below the limit of detection was too high to provide valid result. ^b^ #<LOD, and ƛCA not provided by The Fourth National Report on Human Exposure to Environmental Chemicals, 2019. Abbreviations: TCPy; 3,5,6-trichloro-2-pyridinol; 3-PBA, 3-Phenoxybenzoic Acid; cis- and trans-DCCA, cis/trans-3-(2,2-dichlorovinyl)-2,2-dimethylcyclopropane carboxylic acid; and λCA, λ cyhalothrin.

**Table 3:** Adjusted associations for natural log-transformed chlorpyrifos metabolite TCPy (3,5,6-trichloro-2-pyridinol) and pyrethroid metabolite 3-PBA (3-Phenoxybenzoic acid) concentrations in urine with general intellectual ability measured by the Woodcock–Muñoz Battery, and domains of executive functions measured by the CANTAB in the multiple imputed samples (*n* = 241).

	Models of Single Metabolites, β [95% CI] ^1^		Models of Both Metabolites Together, β [95% CI] ^1,2^	
Outcome	TCPy	3-PBA	TCPy	3-PBA
General intellectual ability	−0.82 [−3.10, 1.45]	−0.95 [−2.89, 0.99]	−0.55 [−3.22, 2.12]	−0.84 [−3.12, 1.43]
IED, stages completed	0.01 [−0.002, 0.02]	0.03 [0.01, 0.05]	0.002 [−0.02, 0.02]	0.03 [0.004, 0.05]
IED, total trials	−0.04 [−0.07, −0.01]	−0.07 [−0.10, −0.04] *	−0.02 [−0.05, 0.02]	−0.07 [−0.10, −0.03] *
IED, total errors	−0.06 [−0.11, −0.007]	−0.12 [−0.18, −0.07] *	−0.02 [−0.08, 0.04]	−0.12 [−0.18, −0.07] *
IED, predimensional errors	0.004 [−0.07, 0.08]	−0.05 [−0.13, 0.03]	0.02 [−0.05, 0.10]	−0.05 [−0.13, 0.02]
IED, postdimensional errors	−0.11 [−0.17, −0.06] *	−0.11 [−0.19, −0.02]	−0.08 [−0.15, −0.02]	−0.09 [−0.17, −0.01]
SSP, span length	−0.02 [−0.07, 0.02]	0.01 [−0.02, 0.05]	−0.03 [−0.07, 0.01]	0.02 [−0.02,0.06]
SOC, problems solved	0.01 [−0.05, 0.07]	0.01 [−0.03, 0.06]	0.01 [−0.07, 0.08]	0.01 [−0.05, 0.07]

^1^ Models adjusted for age at assessment (months), sex, season (fall, winter, spring, summer) blood lead (<to 3.5 μg/L and ≥3.5 μg/L), school clusters, hemoglobin, and HOME inventory scores. ^2^ Models include both pesticide metabolites as predictors of cognitive performance. * *p* < 0.05 with Holm–Bonferroni correction. Abbreviations: SSP: Spatial Span, SOC: Stockings of Cambridge, IED: Intra-Extra Dimensional Set Shift.

**Table 4:** Adjusted associations for natural log-transformed chlorpyrifos metabolite TCPy (3,5,6-trichloro-2-pyridinol) and pyrethroid metabolite 3-PBA (3-Phenoxybenzoic acid) concentrations in urine with general intellectual ability measured by the Woodcock–Muñoz Battery, and domains of executive functions measured by the CANTAB in the multiple imputed samples (*n* = 241) stratified by blood lead levels (<3.5 and ≥3.5 μg/dL).

	Models of Single Metabolites, β [95% CI] ^1^				Models of Both Metabolites Together, β [95% CI] ^1,2^			
	TCPy		3-PBA		TCPy		3-PBA	
	BLL < 3.5	BLL ≥ 3.5	BLL < 3.5	BLL ≥ 3.5	BLL < 3.5	BLL ≥ 3.5	BLL < 3.5	BLL ≥ 3.5
General intellectual ability	−1.38	−0.31	−1.42	−0.51	−1.18	0.06	−1.34	−0.36
	[3.84, 1.08]	[−5.64, 5.01]	[−3.52, 0.68]	[−3.15, 2.12]	[−3.25, 0.89]	[−5.98, 6.12]	[−3.67, 0.99]	[−3.31, 2.59]
IED, stages completed	0.02	0.01	0.03 *	0.02	0.01	−0.003	0.04	0.02
	[0.003, 0.03]	[−0.06, 0.06]	[0.02, 0.05]	[−0.02, 0.06]	[−0.01, 0.02]	[−0.03, 0.02]	[0.01, 0.06]	[−0.01, 0.04]
IED, total trials	−0.08 *	−0.03	−0.08 *	−0.05	−0.03	−0.01	−0.08 *	−0.05
	[−0.13, −0.04]	[−0.07, 0.01]	[−0.12, −0.04]	[−0.09, −0.01]	[−0.05, −0.01]	[−0.06, 0.04]	[−0.13, −0.04]	[−0.09, −0.01]
IED, total errors	−0.08 *	−0.04	−0.16 *	−0.09	−0.04	0.0004	−0.16 *	−0.09
	[−0.26, −0.04]	[−0.11, 0.02]	[−0.22, −0.09]	[−0.21, 0.01]	[−0.08, 0.003]	[−0.08, 0.08]	[−0.25, −0.07]	[−0.16, −0.01]
IED, predimensional errors	−0.001	0.01	−0.05	−0.05	0.02	0.03	−0.06	−0.05
	[−0.08, 0.07]	[−0.07, 0.10]	[−0.15, 0.05]	[−0.14, 0.04]	[−0.05, 0.08]	[−0.05, 0.11]	[−0.15, 0.04]	[−0.14, 0.03]
IED, postdimensional errors	−0.21	−0.04	−0.14	−0.08	−0.08	−0.07	−0.12	−0.06
	[−0.33, −0.08]	[−0.11, 0.03]	[−0.28, −0.0005]	[−0.15, 0.002]	[−0.15, −0.02]	[−0.16, 0.01]	[−0.26, 0.02]	[−0.14, 0.02]
SSP, span length	−0.02	−0.02	0.02	0.009	−0.03	−0.04	0.03	0.01
	[−0.06, 0.01]	[−0.07, 0.01]	[−0.02, 0.06]	[−0.03, 0.05]	[−0.07, 0.002]	[−0.09, 0.01]	[−0.01, 0.06]	[−0.03, 0.06]
SOC, problems solved	0.01	0.01	0.01	0.01	0.01	0.01	0.01	0.01
	[−0.06, 0.08]	[−0.05, 0.07]	[−0.04, 0.07]	[−0.04, 0.07]	[−0.07, 0.08]	[−0.07, 0.08]	[−0.05, 0.07]	[−0.06, 0.08]

^1^ Models adjusted for age at assessment (months), sex, season (fall, winter, spring, summer), school clusters, hemoglobin, and HOME inventory scores ^2^ Models include both pesticide metabolites as predictors of cognitive performance. * *p* < 0.05 with Holm–Bonferroni correction. Abbreviations: SSP: Spatial Span, SOC: Stockings of Cambridge, IED: Intra-Extra Dimensional.

## Data Availability

The data are not publicly available due to sensitive participant information. The data presented in this study are available on request from the corresponding author.

## Supplementary Materials

The following are available online at https://www.mdpi.com/article/10.3390/ijerph20075288/s1, Table S1: Spearman Coefficients (ρ) for creatinine-adjusted pesticide metabolites in children aged ~7 years from Montevideo; and Table S2: Adjusted associations for natural log-transformed chlorpyrifos metabolite TCPy (3,5,6-trichloro-2-pyridinol) and pyrethroids metabolite 3-PBA (3-Phenoxybenzoic acid) concentrations in urine with general intellectual ability measured by the Woodcock-Muñoz Battery, and domains of executive functions measured by the CANTAB in the non-imputed sample.
