# Supplementary material for: Pyrethroid and Chlorpyrifos Pesticide Exposure, General Intellectual Abilities, and Executive Functions of School Children from Montevideo, Uruguay
Source: Int J Environ Res Public Health. 2023 Mar 28;20(7):5288. doi: 10.3390/ijerph20075288 (PMC10093823; doi:10.3390/ijerph20075288)
Supplement: Supplementary file 1 [file ijerph-20-05288-s001.zip › Supplemental Table S1.pdf]

Table S1. Spearman Coefficients (ρ) for creatinine-adjusted pesticide metabolites in children aged ~ 7 years from Montevideo (n = 241).

	Pyrethroids (ng/mg)				Chlorpyrifos (ng/mg)
	λCA	Cis-DCCA	Trans-DCCA	3-PBA	TCPy
Cis-DCCA	-0.02	-	**0.91****	**0.68****	0.11
Trans-DCCA	-0.03	**0.91****	-	**0.73****	0.12
3-PBA	-0.004	**0.68****	**0.73****	-	**0.17***
TCPy	**0.29****	0.11	0.12	**0.17***	-

Pesticide metabolite abbreviations: TCPy, 3,5,6-trichloro-2-pyridinol; 3-PBA, 3-Phenoxybenzoic Acid; cis- and trans-DCCA, cis/trans-3-(2,2-dichlorovinyl)-2,2-dimethylcyclopropane carboxylic acid; and λCA, λ- cyhalothrin; ** $p < 0.0001$, * $p < 0.05$.
