# Supplementary material for: Pyrethroid and Chlorpyrifos Pesticide Exposure, General Intellectual Abilities, and Executive Functions of School Children from Montevideo, Uruguay
Source: Int J Environ Res Public Health. 2023 Mar 28;20(7):5288. doi: 10.3390/ijerph20075288 (PMC10093823; doi:10.3390/ijerph20075288)
Supplement: Supplementary file 1 [file ijerph-20-05288-s001.zip › Supplemental Table S2.pdf]

Table S2. Adjusted associations for natural log-transformed chlorpyrifos metabolite TCPy (3,5,6-trichloro-2-pyridinol) and pyrethroids metabolite 3-PBA (3-Phenoxybenzoic acid) concentrations in urine with general intellectual ability measured by the Woodcock-Muñoz Battery, and domains of executive functions measured by the CANTAB in the non-imputed sample (N = 222).

	**Models of single metabolites, β [95% CI] [1]**		**Models of both metabolites together, β [95% CI] [1,2]**	
**Outcome**	**TCPy**	**3-PBA**	**TCPy**	**3-PBA**
General intellectual ability	-0.24 [-2.83,2.34]	-1.28 [-3.3,0.74]	0.18 [-2.47,2.84]	-1.32 [-3.39,0.74]
IED, stages completed	0.01 [-0.01,0.02]	0.02 [0.0001,0.05]	-0.002 [-0.02,0.01]	0.02 [-0.001,0.05]
IED, total trials	-0.03 [-0.06,-0.003]	**-0.06 [-0.10,-0.02]***	-0.01 [-0.05,0.02]	-0.06 [-0.10,-0.02]
IED, total errors	-0.04 [-0.10,0.01]	**-0.11 [-0.18,-0.05]***	-0.005 [-0.06,0.05]	**-0.11 [-0.18,-0.04]***
IED, pre-dimensional errors	0.01 [-0.07,0.10]	-0.05 [-0.13,0.03]	0.03 [-0.05,0.11]	-0.06 [-0.13,0.02]
IED, post-dimensional errors	**-0.11 [-0.17,-0.05]***	-0.10 [-0.18,-0.01]	-0.08 [-0.16,-0.0004]	-0.08 [-0.17,0.01]
SSP, span length	-0.02 [-0.07,0.02]	0.01 [-0.03,0.05]	-0.03 [-0.07,0.01]	0.02 [-0.02,0.05]
SOC, problems solved	0.01 [-0.04,0.06]	0.003 [-0.04,0.04]	0.01 [-0.06,0.06]	0.002 [-0.05,0.05]

[1] Models adjusted for age at assessment (months), sex, season (fall, winter, spring, summer) blood lead (< to 3.5 ug/L and ≥ 3.5 µg/L), school clusters, hemoglobin, and HOME inventory scores. [2] Models include both pesticide metabolites as predictors of cognitive performance. * $p < 0.05$ with Holm-Bonferroni correction. Abbreviations: SSP: Spatial Span, SOC: Stockings of Cambridge, IED: Intra-Extra Dimensional Set Shift.
